# Effect of child health status on parents’ allowing children to participate in pediatric research

**DOI:** 10.1186/1472-6939-14-7

**Published:** 2013-02-15

**Authors:** Jérémy Vanhelst, Ludovic Hardy, Dina Bert, Stéphane Duhem, Stéphanie Coopman, Christian Libersa, Dominique Deplanque, Frédéric Gottrand, Laurent Béghin

**Affiliations:** 1 Centre d’Investigation Clinique, CIC-PT-9301-Inserm-CH&U, Lille, 59037, France; 2 Unité Inserm U995 & Université Lille Nord de France, Lille, France; 3 Comité de Protection des Personnes Nord Ouest IV, Lille, France; 4 Département de Pharmacologie, Faculté de Médecine, Université Lille Nord de France, Lille, France

**Keywords:** Ethics, Pediatric research, Parents’ acceptance, Motivation

## Abstract

**Background:**

To identify motivational factors linked to child health status that affected the likelihood of parents’ allowing their child to participate in pediatric research.

**Methods:**

Parents were invited to return their completed questionnaires anonymously to assess motivational factors and factors that might improve participation in pediatric research.

**Results:**

Of 573 eligible parents, 261 returned the completed questionnaires. Of these, 126 were parents of healthy children (group 1), whereas 135 were parents of sick children who were divided into two groups according to the severity of their pathology, *i*.*e*., 99 ambulatory children (group 2) and 36 nonambulatory children (group 3). The main factor motivating participation in a pediatric clinical research study was “direct benefits for their child” (87.7%, 100%, and 100% for groups 1, 2, and 3, respectively). The other factors differed significantly between the three groups, depending on the child’s health status (all *p* < 0.05). Factors that might have a positive impact on parental consent to the participation of their child in a pediatric clinical research study differed significantly (χ^2^ test, all *p* ≤ 0.04), depending on the child’s health status. The main factor was “a better understanding of the study and its regulation” for the healthy children and ambulatory sick children groups (31.2% and 82.1%, respectively), whereas this was the third factor for the nonambulatory sick children group (50%).

**Conclusions:**

Innovative strategies should be developed based on a child’s health status to improve information provision when seeking a child’s participation in pediatric research. Parents would like to spend more time in discussions with investigators.

## Background

There is an increasing need in pediatrics to perform age-specific clinical trials to improve drug safety and to ensure that the best medical treatment is available to children [1]. Therefore, the requirement for high-quality pediatric clinical research studies is growing in industrial and academic organizations [2-6]. In this context, regulatory and ethical considerations have led to the development of specific pediatric regulations and guidelines [7-9].

Recruitment remains one of the main difficulties in clinical research [10,11]. Good Recruitment Practices were published by Bachenheimer and Brescia, but these guidelines fail to consider adequately the responsibilities of parents who care for their children, particularly the following issues [12,13]: (*i*) the participation of children requires consent from their parents, (*ii*) children are a vulnerable population, and invasive procedures and pain should be limited as much as possible, (*iii*) the time constraints of studies may have consequences for the family structure and organization (*e*.*g*., jobs, school attendance, and siblings), and (*iv*) parents have a negative perception of participation in medical research [14-20]. Thus, Lasagna’s law (*i*.*e*., the “number of patients in the predictive pool always exceed those eligible, which again exceeds those who consent during the recruitment period of the study”) may have a greater impact on pediatric studies than on adult clinical studies. The involvement of parents in the health care of their child and the parental perception of pediatric clinical research studies may also affect the study design, particularly with invasive studies [21-23].

Thus, investigators need to present parents and children with a well-balanced view of the risks and benefits of studies and to provide explanations for any procedures involved in pediatric clinical research studies. The majority of research into the participation of children and adolescents in research has focused on the child’s understanding of pediatric clinical research studies [24]. Some previous studies have addressed the motivation of parents during the enrolment of their children in pediatric clinical research studies, but there have been no previous investigations of whether the health status of their child might affect parental consent to involvement in pediatric clinical research studies [8,25-28]. We hypothesized that the parents of sick children who had grown up with the rules and regulations of the health care system may have a specific perception of pediatric clinical research studies that could affect their motivation when making a decision about participation in pediatric clinical research studies [16].

The primary aim of this study was to identify motivational factors linked to child health status that affected the likelihood of parents’ allowing their child to participate in a pediatric clinical research study. We also aimed to identify key factors that might increase the probability of parents’ allowing their child to participate in a pediatric clinical research study.

## Methods

### Participants

**Figure 1:**
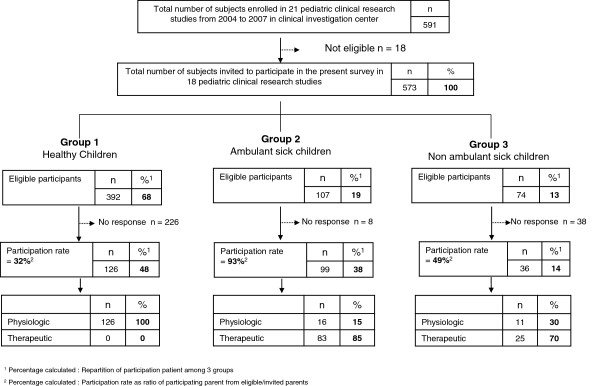
Summary of recruitment process and ratio of physiologic/therapeutic pediatric clinical research study.

Between 2004 and 2007, 22 pediatric clinical research studies were conducted at Lille Clinical Investigation Center (Lille University Hospital; CIC-PT-9301-Inserm-CH&U, Lille, France), and 18 met the selection criteria. The selection criteria were: (*i*) pediatric clinical research study conducted between 2004 and 2007, and (*ii*) child aged between 1 and 18 years. The exclusion criteria were: (*i*) pediatric clinical research studies involving neonates hospitalized in the intensive care unit, (*ii*) children enrolled in oncology pediatric clinical research studies, who were considered to be a highly specific group of patients with an immediate, potentially poor outcome, (*iii*) babies enrolled in industrial milk formula studies, and (*iv*) other studies involving children aged less than one year. A summary of the recruitment process is shown in Figure 1. The children were divided into three groups according to their disease severity to assess their participation in pediatric research. This classification was clinically relevant because it can assess the severity of diseases based on their impact on the quality of life, and it can also be used as an indicator of functional ability. Thus, we decided to separate the group of sick children into two subgroups because we hypothesized that the severity of the pathology had different effects on participation in clinical research [29]. **Group 1** contained healthy children (n = 392; **68%**). **Group 2** (n = 107; **19%**) contained children with diabetes, asthma, cystic fibrosis, phenylketonuria, and gastroesophageal reflux disease. **Group 3** (n = 74; **13%**) contained children with severe handicaps such as myopathy and encephalopathy. Physiological pediatric clinical research studies included 100% healthy children, whereas 85% and 70% of the children in the sick groups were included in therapeutic pediatric clinical research studies, *i*.*e*., ambulatory and nonambulatory sick children, respectively.

This study was approved by the local research ethics committee (Comité de Protection des Personnes Nord Ouest IV, Lille, France). This study did not involve any interventions and it was retrospective; thus, informed consent was not required according to French human research regulations. The answers provided by parents were anonymous and confidential, and so informatic personal data regulatory authority approval was not necessary to conduct this survey.

### Questionnaire

The questionnaire was written by a focus group of highly skilled professionals (CIC 9301 INSERM-CHU, Lille, France) with experience of pediatric clinical research studies, including a pediatrician, a physician, a member of the local Ethical Review Board, a psychologist, a clinical research assistant/scientist, a study coordinator, and a study nurse. Before data collection, an initial pretest questionnaire was completed by a sample of 10 parents. This pretest was conducted to evaluate the clarity, comprehensiveness, and acceptability of each question, and the questionnaire length. A second pretest (pilot study) was completed by a second sample of 15 parents to assess the quality and response rate for each question. Questions were deleted if they were completed by less than 80% of subjects. After each step, appropriate changes were made to produce the final questionnaire.

The final questionnaire was divided into three parts: **part one**: demographic and social information, and the child’s medical condition; **part two**: motivational factors; and **part three**: factors that could improve pediatric clinical research study participation. The demographic information (part one) comprised the age of the participant, the age of the child, and the parental education level. The parental education level was reported using four categories; *i*.*e*., pre-primary school level (score = 1) to high school (tertiary) level (score = 4), according to the International Standard Classification of Education structure (http://www.ins.unesco.org/publications/ISCED97). In the current analysis, the two lower classes were merged into one (*i*.*e*., pre-primary education and primary education) to give the following classification: low, medium, and high.

Questions concerning motivational factors (part two) (Additional file 1: Annex 1) were classified into four categories: (*i*) understanding the study and its regulation, (*ii*) direct benefits to the parent’s own child of participating in the study, (*iii*) benefits to the general population, and (*iv*) low risk to the child of participating in the study. The answers to these questions were a simple binary choice; *i*.*e*., a “yes” or “no” format.

Questions about factors that might improve parental consent to involvement in pediatric clinical research studies (part three) (Additional file 1: Annex 1) were also divided into four sections: (*i*) understanding the study and its regulation, (*ii*) direct benefits to the parent’s own child, (*iii*) low risk to the children, and (*iv*) the modalities whereby information was communicated about the study. The answers to these questions were a simple binary choice; *i*.*e*., a “yes” or “no” format.

A rank order was defined for the two topics and the methodology was as follows. 1. Asking a list of questions in a random order with a “yes/no” reply format. 2. Regrouping the questions depending on the specific items we wanted (four questions for each item) to address. For each parent, we calculated the percentages of “yes” answers and ranked the item as positive if it received 3/4 “yes” replies to these questions (but negative if it received ≤ 2). 3. We pooled all of the results to determine the global percentage of agreement or disagreement at the group level.

Each questionnaire was introduced by an informative letter, which explained the objectives of the survey, and a guarantee that the data would remain strictly anonymous/confidential.

### Survey procedure

After completing a pediatric clinical research study, the investigator invited parents to answer a set of questions concerning their motivational factors and other factors that might improve pediatric clinical research study participation. The questionnaires were completed after the parents and their children returned to their homes. The parents were invited to return their answers anonymously via the postal system. The replies were anonymous so reminders were not possible.

### Statistics

#### Sample size calculation

The requisite sample size was calculated using data from Van Stuijvenberg *et al*. [30]. Van Stuijvenberg *et al*. reported that the major reason (32%) why parents of sick children agreed that their children could participate in a pediatric clinical research study was a benefit to their own child. No previous data were available to allow a comparison between healthy children and different groups of sick children (ambulatory *vs* nonambulatory), and so we calculated the sample size using healthy *vs* a single group of sick children. We assumed that the number of consecutively healthy children recruited would be twice the number of sick children. If we consider a difference of 30% between groups (62% for healthy *vs* 32% for sick children), with 80% power and an alpha risk of 5%, the required sample size would be 64 healthy children and 32 sick children. Our objective was to detect the difference between healthy children and each group of sick children (ambulatory and nonambulatory); therefore, the size of each group of sick children was set to ≥ 32.

#### Statistical analysis

The data were analyzed using the Statistical Package for the Social Sciences, version 11.5 for Windows (SPSS Inc., Chicago, IL, USA). Student’s *t*-test was used to compare the characteristics of the parents and children. The results of the questionnaires were expressed as the percentages of participating parents who answered “yes” or “no” to each item. The χ^2^ test was used to compare differences in the response rates for the healthy and sick groups in terms of the motivational factors and improvement factors. A *p* value of < 0.05 was considered significant.

## Results

### Participation rate and main characteristics of the participating parents

Of 573 eligible parents, 261 questionnaires were completed and returned, giving an overall participation rate of 45.5%. Among this population, 126 (48%) were parents of healthy children (group 1), 99 (38%) were parents of ambulatory children (group 2), and 36 (14%) were parents of nonambulatory children (group 3), and these proportions differed from those observed initially in the target population (*i*.*e*., 68%, 19%, and 13%, respectively; χ^2^ test, *p* < 0.01). The participation rate was significantly different between the three groups (χ^2^ test, *p* < 0.01). Parents of ambulatory sick children had the highest participation rate (93%). The parents who returned the questionnaires answered an average of 98.5% of the questions.

The age characteristics of the participating parents and children did not differ between the three groups (Table 1). However, the majority of the participating parents were the children’s mothers (> 82%). The parental education level was different between the groups (χ^2^ test, *p* = 0.004). Table 1 also shows that 25% of ambulatory and 26% of nonambulatory sick children had participated in a previous pediatric clinical research study whereas only 10% of healthy children had participated previously (*p* = 0.83). Moreover, 79% of the parents of ambulatory sick children and 87% of the parents of nonambulatory sick children had already heard about pediatric clinical research studies compared with 52% of the parents of healthy children (*p* = 0.002).

**Table 1:** Main characteristics of participating parents and children analysed in this survey

	**Group 1**	**Group 2**	**Group 3**	**P**^**1**^
	**Healthy children**	**Ambulant sick children**	**Non ambulant sick children**	
N (*%*)	48.3	37.9	13.8	
Age of participating parent (mean *year* ± SD)	42.1 ± 6.2	39.3 ± 5.1	38.5 ± 3.7	0.91*
Age of child (mean *year* ± SD)	12.3 ± 1.9	9.8 ± 3.4	10.1 ± 3.5	0.82*
Gender of participating parent (*%*)				
Mother	82	93	90	0.80**
Father	18	7	10	
Parental education level (*%*)				
Lower	32	31	49	
Medium	29	47	29	0.004**
High	39	22	22	
Children having previously participate to a study (*%*)	10	25	26	0.83**
Children having already heard about clinical research study (*%*)	52	79	87	0.002**

^*^ ANOVA test ; ^**^ χ^2^ Test.

### Motivational factors

The main factor motivating participation in a pediatric clinical research study (Table 2) was “Direct benefits for their child” (87.7%, 100%, and 100% for groups 1, 2, and 3, respectively). The other factors differed significantly between the three groups, depending on the child health status (all *p* < 0.05).

**Table 2:** Motivation factors from parents to accept their child participating in pediatric clinical research study

	**Group 1**	**Group 2**	**Group 3**	P^*^
	**Healthy children**	**Ambulant sick children**	**Non ambulant sick children**	
	***n = 126***	***n = 99***	***n = 36***	
	*(%*^*1*^*)*	*(%*^*1*^*)*	*(%*^*1*^*)*	
Direct benefits to the parent’s own child of participating in the study	85.7	100.0	100.0	0.88
Benefits to the general population	93.6	87.2	27.6	**0.001**
Low risk to the child of participating in the study	100.0	60.8	70.2	**0.04**
Understanding the study and its regulation	66.7	57.4	13.8	**0.001**

^1^ Percentage expressed as parent who declared “yes” to the set of question about the item concerned.

* χ^2^ Test.

The main reason for parents’ consenting to the involvement of their healthy children was a significantly “low risk” of participating in a pediatric clinical research study (100%). General benefit to science and health was the second motivational factor for the healthy group and ambulatory sick children, whereas the low risk to the child of the pediatric clinical research study was the second choice for nonambulatory sick children. The least important factor was understanding the study and its regulation.

### Improvement factors

Factors that might have a positive impact on parental consent to the participation of their child in a pediatric clinical research study (Table 3) differed significantly (χ^2^ test, all *p* ≤ 0.04), depending on the child’s health status. The main factor was “a better understanding of the study and its regulation” for the healthy children and ambulatory sick children groups (31.2% and 82.1%, respectively), whereas this was the third factor for the nonambulatory sick children group (50%).

**Table 3:** Factors that might have a positive impact on parents’ acceptance for their child participating in pediatric clinical research study

	**Group 1**	**Group 2**	**Group 3**	P^*^
	**Healthy children**	**Ambulant sick children**	**Non ambulant sick children**	
	***n = 126***	***n = 99***	***n = 36***	
	*(%*^*1*^*)*	*(%*^*1*^*)*	*(%*^*1*^*)*	
Understanding of the study and its regulation	31.2	82.1	50.0	**0.001**
Direct benefits to the parent’s own child	12.7	19.0	20.0	**0.04**
Low risk to the children	13.5	77.4	60.0	**0.001**
The modalities whereby information was communicated about the study	19.2	63.1	55.0	**0.001**

^1^ Percentage expressed as parent who declared “yes” to the set of question about the item concerned.

* χ^2^ Test.

The modality used for communicating information about the study was the second factor for the healthy and nonambulatory sick groups, whereas a low risk of participating in the study was the second factor for the sick ambulatory group. The least important factor was direct benefit to their own children for both groups.

In addition, we found that 13%, 29%, and 40% of the parents of healthy, ambulatory, and nonambulatory sick children, respectively, would have liked to spend more time with investigators discussing the trial.

## Discussion

Children have been the subjects of medical research for hundreds of years, but the present survey is the first to be conducted with adequate statistical power (the computed *a posteriori* power ranged from 80% to > 95%) that has attempted to identify factors influencing parental consent to the participation of their child in a pediatric clinical research study, according to their health status [14]. The main results of this study were the significant differences in the factors motivating parents and the improvement factors among the three groups. The rank orders of the items in the two questionnaires also differed between the groups.

### Participation rate

As mentioned previously, no reminders were sent in this survey because the survey was completely anonymous and we wanted to determine the actual participation rate without the influence of a reminder. The overall participation rate of our survey was 45.5%, which was comparable to other surveys conducted via the postal service [31].

The health status of the eligible participants was significantly different from that of the analyzed participants (χ^2^ test, *p* < 0.001). This was mainly due to the high participation rate among parents of sick ambulatory children (the participation rate was 93% in this group; Figure 1). The groups studied contained different numbers (much smaller in the nonambulatory group) and the participation rates also differed between groups (much higher in sick children), which could have affected the results of our study. However, these differences were expected because sick children are less common than healthy ones but their parents are more enthusiastic/interested in participating in a clinical trial because they expect direct benefits for their children. However, three reasons unrelated to direct health benefits could also have influenced the motivation of parents: (*i*) parents with experience of the health care system were more willing to allow their child to be enrolled in a pediatric clinical research study, (*ii*) some sick children had already participated in a pediatric clinical research study, and (*iii*) some parents of sick children were already well informed about pediatric clinical research studies by active patient associations or specific organizations that promote clinical research participation (in France, for example, the French Myopathy Association: http://www.afm-telethon.fr; the Cystic Fibrosis Association:http://www.vaincrelamuco.org; and http://www.notre-recherche-clinique.fr/) [32]. Most of the participating parents were the children’s mothers, which agreed with other surveys of parents’ opinions of pediatric clinical research studies [33].

The question of low risk is also crucial. We found that 100% of the parents of healthy children considered that low risk was the most important factor. This percentage was lower among parents of sick children (60.8% for ambulatory and 70.2% for nonambulatory sick children), so it was possible to compare the item “low risk” using younger and older parents (median age = 40 years). We found that 64% of younger parents considered that “low risk” was the most important factor compared with 36% of older parents (χ^2^ test, *p* = 0.03). This was because older, more experienced parents perceived the risks as lower than younger parents did [34,35].

### Motivational factors

Irrespective of the severity of the pathology, our survey found that the main reason for parents’ consenting to their child participating in a pediatric clinical research study was direct benefit to their own child. This agreed with previous parental assessments of the benefit/risk ratio for their child [30,36]. Altruistic motivation was the second reason for the involvement of healthy children and those with low pathology severity. A low risk of participating in a study was also a major reason in all groups. Our results agree with those of Rothmier *et al*., Varma *et al*., and Nabulsi *et al*., where the most important factor was the benefit to a parent’s own child, while the secondary factor was altruistic motivation [23,25,27]. However, some surveys have found that the main reason was altruistic motivation or a low perceived risk [18,26,37,38]. These contradictory results were probably because: (*i*) the study populations were very different from our survey, and (*ii*) many other factors can influence the perception of a pediatric clinical research study, such as the parent’s personality, religion, ethnicity, socioeconomic status, and level of education [39-43].

### Improvement factors

Similar to the motivational factors, the factors that might improve the likelihood of parents’ consenting to their child’s participation in a pediatric clinical research study were different between the groups, and they were ranked differently. Indeed, “Understanding of the study and its regulation” was a minor motivational factor, whereas it was the most important improvement factor. “Modalities of information” was a major item that could have a positive impact on parental consent. This item was ranked first because most parents would like to spend more time with physicians discussing pediatric clinical research studies. Our survey found that 13%, 29%, and 40% of the parents of healthy, ambulatory, and nonambulatory sick children, respectively, would like to spend more time with investigators discussing pediatric clinical research studies. The following statement concerning the consent process is found in GCP/ICH E6/4.8.7: the “investigator [] should provide the subject’s or the subject’s legally acceptable representative (in the case of this survey, it was parents) ample time and opportunity to inquire about details of the trial and to decide whether or not to participate in the trial (EMEA)” [9]. The same observation (*i*.*e*., that parents would like to spend more time with physicians) was also reported by Snowdon *et al*. [44]. Similar suggestions were recorded in other adult and pediatric surveys [45-47]. Parents would prefer to discuss pediatric clinical research studies with investigators directly and to receive greater reassurance before making a decision rather than reading and signing lengthy and complex information/consent forms, which have increased in length over the years [48]. Previous studies have shown that subjects who were well-informed and who received adequate information about a pediatric clinical research study (*e*.*g*., the study’s aim, expected benefits, potential adverse events and discomfort, study design, regulation, and study procedure) were more prepared to participate in a pediatric clinical research study, with better compliance and a reduced premature withdrawal rate [49].

Our survey showed that safety was the main concern for the parents of healthy children, whereas the direct benefit of pediatric clinical research studies was the main reason for the parents of sick children, and this was the major motivation for parents’ consenting to their children being involved in pediatric clinical research studies. The main improvement factor was clearly that investigators should spend more time discussing pediatric clinical research studies with parents. Indeed, several institutional research centers have already constructed pediatric clinical research centers with specific research teams that include pediatricians, physicians, study nurses, and clinical research assistants, who aim to improve the performance quality of pediatric clinical research studies (http://www.cic-pediatriques.fr) [50,51]. These centers could allow parents to have more personal discussions with staff devoted to pediatric clinical research studies, which appears to be the best method of communicating complex information about pediatric clinical research studies [52-55]. The promotion of pediatric clinical research studies should also consider diverse aspects of pediatric clinical research studies, and the best approaches should be learned during pediatric resident fellowships, as proposed by Massimo *et al*. and Roth [56,57]. Finally, pediatric investigators and/or pediatric clinical research study sponsors should provide improved and innovative pediatric clinical research study information strategies with specific material, as proposed by Wulf *et al*. [24,58]. Previous successful innovations have included: (*i*) videotape recordings, (*ii*) modified consent forms containing figures/pictures, and (*iii*) a specific handbook that improves knowledge and perceptions of clinical trials [59-61]. Recently, the StaR Child Health group developed evidence-based guidance for the design, conduct, and reporting of pediatric clinical research studies, including recruitment and informed consent in order to reach “agreement on how to best recruit children in an efficient and ethical manner” [62].

## Conclusions

Our survey demonstrated that a child’s health status and pathology severity are novel factors that investigators should consider when seeking a parent’s consent for child participation in a pediatric clinical research study. Improvements could be made to current practices based on a child’s health status and pathology severity to increase the likelihood of parental consent, which may increase the participation rate in pediatric clinical research studies. The main lesson of this survey is that parents would like to spend more time in discussions with investigators before enrolling their child in a pediatric clinical research study.

## Abbreviations

GRP: Good recruitment practices; ISCED: International standard classification of education; GCP/ICH: Good clinical practice/ international conference of harmonization; EMEA: European medicines agency.

## Competing interests

All authors do not have any competing interests.

## Authors’ contributions

LB, LH, FG, CL, DD conceived the idea. JV and DB wrote the first and subsequent drafts. SC and SD helped developed the ideas. All authors read and approved the final manuscript.

## Pre-publication history

The pre-publication history for this paper can be accessed here:

http://www.biomedcentral.com/1472-6939/14/7/prepub

## Supplementary Material

Additional file 1 **Annex 1.** Questionnaire on motivational factors and the factors that might improve parental consent. (DOC 70 kb) Click here for file
