# Supplementary material for: Effect of child health status on parents’ allowing children to participate in pediatric research
Source: BMC Med Ethics. 2013 Feb 15;14:7. doi: 10.1186/1472-6939-14-7 (PMC3582492; doi:10.1186/1472-6939-14-7)
Supplement: Additional file 1 — Annex 1. Questionnaire on motivational factors and the factors that might improve parental consent. (DOC 70 kb) [file 1472-6939-14-7-S1.doc]

Annex 1: Questionnaire on motivational factors and the factors that might improve parental consent.

| **Motivational factors** | | |
| --- | --- | --- |
| You decided to allow your child to participate in the study because… |  |  |
| ***Understanding the study and its regulation*** | ***Yes*** | ***No*** |
| A detailed information form was provided |  |  |
| I was informed that I had the right to withdraw my child from the study whenever I wanted |  |  |
| The study was well explained |  |  |
| Confidentiality was preserved |  |  |
| ***Direct benefits to the parent’s own child when participating in the study*** |  |  |
| There was a potential health benefit for my child if they participated in the study |  |  |
| Participating in the study led to a better follow-up compared with the usual care |  |  |
| My child received an innovative treatment |  |  |
| The health status of my child was monitored closely |  |  |
| ***Benefits to the general population*** |  |  |
| The study could improve the future health care for the disease involved |  |  |
| The study could help other children |  |  |
| The topic of the study was interesting |  |  |
| The study could improve knowledge of the disease |  |  |
| ***Low risk to the child of participating in the study*** |  |  |
| There were no major risks of participating in the study |  |  |
| The study could not hurt my child |  |  |
| I did not receive disturbing information about my child’s safety |  |  |
| There were no immediate adverse effects on my child |  |  |

| **Factors that might improve parental consent** | | |
| --- | --- | --- |
| You would be more likely to allow your child to participate in a study if… |  |  |
| ***Understanding the study and its regulation*** | ***Yes*** | ***No*** |
| There was a longer period for refection |  |  |
| A clear discussion with the investigator was permitted |  |  |
| The study was approved by an ethics committee |  |  |
| Clear information was provided that I had the right to withdraw my child from the study whenever I wanted |  |  |
| ***Direct benefits to the parent’s own child of participating in the study*** |  |  |
| There was a clear understanding that my child would directly benefit from the study |  |  |
| The study contributed to scientific knowledge |  |  |
| There was a gift/indemnity offered for participation |  |  |
| The efficacy of the medication tested in the study had been demonstrated in an adult |  |  |
| ***Low risk to the child of participating in the study*** |  |  |
| There was no randomization |  |  |
| There was no placebo |  |  |
| Several children had already participated in the study |  |  |
| The study medication had been used for a long time by adults before the study began using children |  |  |
| ***The modalities used to communicated information about the study*** |  |  |
| I could choose the times when I attended the hospital |  |  |
| There were fewer administrative documents to complete |  |  |
| The study duration was reduced |  |  |
| There was more time for discussions with the physician/investigator |  |  |
